# Doppler in High-Risk Pregnancy and Its Correlation With Feto-Maternal Outcomes: A Prospective Study

**DOI:** 10.7759/cureus.56751

**Published:** 2024-03-22

**Authors:** Pinki Pinki, Amit Amit

**Affiliations:** 1 Obstetrics and Gynaecology, Maharaja Agrasen Medical College, Agroha, Hisar, IND; 2 Paediatrics, Maharaja Agrasen Medical College, Agroha, Hisar, IND

**Keywords:** high-risk pregnancy, pregnancy-induced hypertension, newborn, ultrasound, doppler

## Abstract

Introduction: In the current times, fetal growth monitoring has become readily available with the help of Doppler ultrasound. Identification of pregnancies that are at risk for perinatal morbidity and mortality has been a primary goal of obstetric care. Doppler study is a fast, non-invasive test that provides significant information about the hemodynamic status of the fetus. It is an efficient diagnostic modality to assess fetal compromise, which helps in timely intervention in high-risk pregnancies for better perinatal outcomes.

Objectives: The objectives of this study were to know the correlation between antenatal Doppler findings and perinatal outcomes, including preterm labor, cesarean section, birth weight, and rate of admission of neonates in the neonatal intensive care unit (NICU). Admission to NICU was taken as the primary outcome.

Methods: This is a tertiary care hospital-based prospective study done at Maharaja Agrasen Medical College, Agroha, India. A hundred high-risk pregnant women with oligohydramnios, intrauterine growth restriction (IUGR), pregnancy-induced hypertension (PIH), diabetes mellitus (DM), anemia, or Rh incompatibility were included. Pregnancies in the first and second trimesters and congenital anomalies in babies were excluded from the study. The patients were examined for a Doppler study of the umbilical artery, fetal middle cerebral artery (MCA), and both maternal uterine arteries. Parameters in the form of a resistance index (RI), pulsatility index (PI), and systolic/diastolic ratio (S/D) of all the arteries were taken.

Details of delivery and fetal outcomes were recorded. Data were correlated with Doppler findings. For comparing categorical data, the chi-square (X^2^) test and Fisher's exact test were performed. The comparison of continuous data between the two groups was done using an independent t-test. All statistical calculations were done using the computer program IBM SPSS Statistics for Windows, version 25 (released 2015; IBM Corp., Armonk, New York, United States).

Results: All the 100 cases with either normal or abnormal Doppler were comparable in terms of maternal age and parity. The prevalence of oligohydramnios was 27% (N = 27), PIH was seen in 20% (N = 20), anemia in 19% (N = 19), IUGR in 12% (N = 12), and oligohydramnios with IUGR in 13% (N = 13). In oligohydramnios, maternal anemia, Rh incompatibility, and DM, Doppler was found to be normal. In PIH, IUGR, and oligohydramnios with IUGR, abnormal Doppler was seen in four (20%), two (16%), and 10 (76%) cases, respectively. Among 84 candidates with normal Doppler, 49 (58%) got delivered by the vaginal route. Out of 16 abnormal Doppler cases, five were vaginally delivered (31%). Among 16 patients with abnormal Doppler, 15 patients, i.e., 93.75%, had low birth weight (LBW, <2.5 kg) (p-value < 0.001), 93.75% (N = 15) delivered before 37 weeks, and 13 (81.25%) newborns were admitted in the nursery.

Conclusion: Abnormal Doppler was associated with an increased rate of low birth weight and admissions to the NICU with no effect on preterm delivery or cesarean section rates. The study strengthens the fact that Doppler studies in mothers can be used to plan the mode of delivery, predict the need for resuscitation, and anticipate the outcome of newborns.

## Introduction

Fetal development is considered to be a complex process resulting from interactions between the inherent growth potential of the fetus and the effect of the intrauterine maternal environment [1]. Now, in the era of the sophisticated modality of real-time ultrasound, antenatal fetal growth monitoring has become readily available, assisting the decline in perinatal morbidity and mortality, and also the approach has future potential [2].

Hypertensive disorders of pregnancy are important causes of maternal and fetal morbidity and mortality worldwide. Pregnancy-induced hypertension (PIH) in women complicates 5-10% of all pregnancies [3,4]. Other than this entity, oligohydramnios, diabetes mellitus (DM) in mothers, anemia, and Rh isoimmunization have also many adverse fetal and maternal outcomes [5]. Intrauterine growth restriction (IUGR) is usually seen as a result of these mentioned high-risk conditions. IUGR is said to be present when the fetal weight is estimated below the 10th percentile with respect to the gestational age. IUGR leads to a pathological growth restriction, which further results in low fetal weight and numerous adverse effects in early childhood [6].

The rationale for suspecting that Doppler imaging may be a useful diagnostic technique for IUGR is based on a chain of reasoning. Increased resistance, when detected in the feeding arteries, leads to decreased velocity of flow, more so during the diastole. This phenomenon leads to a decreased amount of blood flowing through the placenta. The other important aspect is the detection of disproportionate slowing of diastolic flow as compared to systolic flow, leading to elevated Doppler indices. Important indices are the systolic/diastolic ratio and the pulsatility index.

Considering the above facts, this study was conducted to analyze the role of a Doppler study in high-risk pregnancies and strengthen the facts showing the correlation of Doppler findings with maternal and neonatal outcomes.

## Materials and methods

This is a hospital-based prospective study, conducted in the Department of Obstetrics and Gynecology, with collaboration of the Pediatrics department, at Maharaja Agrasen Medical College, Agroha, in North India. One hundred women with high-risk pregnancies attending both the outpatient and emergency departments, from September 2019 to April 2021 (19 months), were included in the study. Approval was obtained from the Institutional Ethical Committee for Human Research with reference number MAMC/Pharma/IEC/19/25.

Inclusion criteria

Pregnancies with IUGR (asymmetrical), diabetes (both gestational and type 2), anemia in mothers, oligohydramnios, Rh isoimmunization, and PIH of all age groups, irrespective of parity, were included in the study. In cases of Rh isoimmunization, all mothers with a negative blood group with at least one baby with a positive blood group and positive indirect Coombs's test were taken.

Exclusion criteria

Mothers with gestation less than 28 weeks, twin pregnancies, congenital fetal anomalies, and who did not give consent for the study were excluded from the study.

The mothers who were included in the study were followed up as per the guidelines, from the date of enrollment to the time of delivery. They were examined on a color Doppler machine with a 3-5 MHz curvilinear probe using color and spectral Doppler. Detailed ultrasonography study, including estimation of maturity (by biparietal diameter, femoral length, and abdominal circumference), liquor assessment, and expected fetal weight, was done. The fetal middle cerebral artery (MCA), umbilical artery, and both the maternal uterine arteries were included in the Doppler study. The pulsatility index (PI), resistive index (RI), and systolic/diastolic ratio (S/D) of all four arteries were taken as parameters for observation and analysis.

Outcome measures

Details were taken about labor, whether induced or spontaneous. The mode of delivery was noted, whether cesarean section (CS) or vaginal delivery (VD). Details of fetal information were recorded in the form of birth weight, intrauterine death (IUD), stillbirth, IUGR, and APGAR score. Neonates were followed for seven days after birth for their admission to the nursery and outcome.

We collected all the information and segregated the data based on Doppler findings, whether normal Doppler or abnormal. Data comprising mode of delivery, birth weight, Doppler indices, and neonatal details were compared with respect to outcomes in both categories.

For comparing categorical data, the chi-square (X^2^) test and Fisher's exact test were performed. The comparison of continuous data between the two groups was done using an independent t-test. All statistical calculations were done using the computer program IBM SPSS Statistics for Windows, version 25 (released 2015; IBM Corp., Armonk, New York, United States. 

## Results

All the cases with either normal or abnormal Doppler were comparable in terms of maternal age and parity. Among 100 patients, the prevalence of oligohydramnios was 27% (N = 27), PIH was seen in 20% (N = 20), anemia in 19% (N = 19), IUGR in 12% (N = 12), and oligohydramnios with IUGR in 13% (N = 13). Prevalence of Rh incompatibility, DM, and PIH with IUGR was 6% (N = 6), 2% (N = 2), and 1% (N = 1), respectively. Table 1 shows the number of patients with abnormal Doppler findings in each high-risk category. In PIH, IUGR, and oligohydramnios with IUGR, abnormal Doppler was seen in four (20%), two (16%), and 10 (76%) cases, respectively.

**Table 1:** Prevalence of high-risk factors and their Doppler findings P-values <0.05 are considered significant. DM: diabetes mellitus, NC: not calculated, IUGR: intrauterine growth restriction, PIH: pregnancy-induced hypertension

High-risk condition	Total number of patients (N = 100)	Patients with normal Doppler (N = 84) and %out of the specific category	Patients with abnormal Doppler (N = 16) and % out of the specific category	p-value (chi-square value)
Oligohydramnios	27	27 (100)	0 (0%)	NC
Anemia	19	19 (100)	0 (0%)	NC
Diabetes mellitus (DM)	2	2 (100)	0 (0%)	NC
IUGR	12	10 (84%)	2(16%)	0.946
Oligohydramnios+ IUGR	13	3 (24%)	10 (76%)	<0.001
PIH	20	16 (80%)	04 (20%)	.585
PIH+IUGR	1	1 (100%)	0 (0%)	NC
RH incompatibility	6	6 (100%)	0 (0%)	NC

In uterine artery findings, 6% (N = 6) of the cases had raised RI, PI, and S/D ratio, and out of them, a persistence notch was present in two cases. MCA Doppler findings with brain sparing, which is considered abnormal, were obtained in 8%(N = 8) of the population. Umbilical artery Doppler with raised RI, PI, and S/D ratio was observed in 12% (N = 12) of the population.

In candidates with normal Doppler, 49 out of 84, and with abnormal Doppler, five out of 16 got delivered by the vaginal route. Among the cases who had vaginal delivery, 9.26% (N = 5) had abnormal Doppler, and among the patients who underwent lower-segment cesarean section (LSCS), 23.9% (N = 11) had abnormal Doppler. This difference was not statistically significant (p-value = 0.0579).

Table 2 shows that out of 16 women with abnormal Doppler, 93.75% (N = 15) delivered before 37 weeks and one delivered after 37 weeks (p-value = 0.183). Among 16 patients with abnormal Doppler, 15 patients, i.e., 93.75%, had low birth weight (LBW, <2.5 kg) (p-value < 0.001). This was statistically significant.

**Table 2:** Correlation of Doppler findings with the period of gestation at delivery and birth weight P-values <0.05 are considered significant. LBW: low birth weight

Outcome variable	Outcome	Number of patients; normal Doppler N = 84	Number of patients; abnormal Doppler N =16	p-value Fisher's exact test
Gestational age at delivery (weeks)	<37	63	15	0.183
	37-41	21	1	
Birth weight	Normal	50	1	<0.001 (Significant)
	LBW	34	15	

Out of 100 newborns, 35 needed admission and 13 out of them had abnormal Doppler findings. Among newborns of 16 women with abnormal Doppler, 13 (81.25%) newborns were admitted at the nursery. Among the babies who were not admitted, 95.38% had normal antenatal Doppler (p-value < 0.001), the difference was statistically significant. Adverse fetal outcomes showed a stronger association with Doppler abnormality of the umbilical artery than with MCA. It was observed that isolated uterine artery abnormality had no statistically significant relation with the neonatal outcome.

## Discussion

This study was primarily done to evaluate whether an abnormal Doppler finding is useful in predicting the neonatal outcome in high-risk pregnancies, including IUGR, oligohydramnios, anemia, DM, PIH, and Rh isoimmunization.

The parity and age of the mother showed no effect on the Doppler findings. Similar results were reported by Manandhar et al. in 2018 with a study done on 60 cases [7]. However, Dalal and Malhotra reported the incidence as 42% in primigravida and 58% in multigravida [8]. Özkan et al. studied the Doppler effects with oligohydramnios and found that the median age of mothers presenting with this entity was 23.7 years (18.1-41 years) [9].

It was observed that in oligohydramnios, maternal anemia, Rh incompatibility, and DM, the Doppler was normal in all cases. However, in PIH, IUGR, and oligohydramnios plus IUGR, abnormal Doppler was seen. In a study conducted by Shah et al. in 2017 on high-risk pregnancies, 43 cases of IUGR fetuses were observed. Among these patients, 38 cases were found to have abnormal Doppler findings, which thereby led to early interventions in these cases [10]. Kramer and Weiner in 1997 also suggested that all cases with IUGR fetuses should be followed up with Doppler studies as soon as possible [11].

This study did not show a significant effect of abnormal Doppler on the rate of vaginal delivery or LSCS. Dalal and Malhotra studied the cases with oligohydramnios, IUGR, and hypertension and reported a more common mode of delivery as vaginal delivery in 68% of cases when the rest underwent LSCS [8].

A significant association was seen between abnormal Doppler and LBW. Sharma et al. reported in their study that pregnancies diagnosed to have IUGR delivered 81.5% of infants with LBW [12]. Manandhar et al. reported almost 28.3% of LBW in IUGR-diagnosed pregnant women [7].

Neonates with abnormal Doppler showed a significantly higher rate of admission at the NICU as compared to the ones with normal Doppler. Dalal and Malhotra also showed similar results [8]. Nalini et al. reported in a study that 35% of neonates with abnormal Doppler flow and only 5% of cases with normal Doppler flow required NICU admissions [13].

Uterine artery Doppler abnormality was not found to have a significant relation with neonatal outcomes. The results of a study, which showed abnormal uterine artery Doppler in 4.5% of the patients at 16-22 weeks, reported a majority of these patients normal at term [14].

Abnormal umbilical artery Doppler has been found to show a significant relationship with low APGAR and admissions in NICU. Another study has reported similar results that umbilical artery PI was the most sensitive and specific for the prediction of IUGR (82.1% and 87%) [15].

The above findings, in terms of various maternal and perinatal outcomes, were analyzed with a 100% follow-up rate. The association of risk factors, like PIH, anemia, and oligohydramnios, with outcomes like IUGR, preterm delivery, and requirement of admission in NICU were correlated with the help of Doppler. The study strengthens the fact that Doppler studies in mothers can be used to plan the mode of delivery, predict the timing of delivery, counsel the parents, and anticipate the outcome of newborns.

A few limitations of this study were also recognized. First, this is a single-center study and ultrasonographic findings may be subjective. A multicentric study could have given better results. Second, only one modality was used to anticipate the outcomes. Certain other tests, such as a non-stress test, if considered simultaneously, could have made the correlation more significant.

## Conclusions

The study showed no correlation of Doppler findings with gestational age at delivery or mode of delivery in high-risk pregnancies. However, the increased rate of LBW and higher rate of admission to NICUs were seen in cases with abnormal Doppler studies in mothers. Doppler studies are therefore helpful in pregnancies with intrauterine growth restriction, PIH, and oligohydramnios.
